# Ventricular stimulus site influences dynamic dispersion of repolarization in the intact human heart

**DOI:** 10.1152/ajpheart.00159.2016

**Published:** 2016-07-01

**Authors:** Neil T. Srinivasan, Michele Orini, Ron B. Simon, Rui Providência, Fakhar Z. Khan, Oliver R. Segal, Girish G. Babu, Richard Bradley, Edward Rowland, Syed Ahsan, Anthony W. Chow, Martin D. Lowe, Peter Taggart, Pier D. Lambiase

**Affiliations:** ^1^Department of Cardiac Electrophysiology, The Barts Heart Center, St Bartholomew's Hospital, London, United Kingdom; and; ^2^Institute of Cardiovascular Science, University College London, London, United Kingdom

**Keywords:** human, whole heart, restitution, APD heterogeneity, dispersion of repolarization

## Abstract

The spatial variation in restitution properties in relation to varying stimulus site is poorly defined. This study aimed to investigate the effect of varying stimulus site on apicobasal and transmural activation time (AT), action potential duration (APD) and repolarization time (RT) during restitution studies in the intact human heart. Ten patients with structurally normal hearts, undergoing clinical electrophysiology studies, were enrolled. Decapolar catheters were placed apex to base in the endocardial right ventricle (RV_endo_) and left ventricle (LV_endo_), and an LV branch of the coronary sinus (LV_epi_) for transmural recording. S1–S2 restitution protocols were performed pacing RV_endo_ apex, LV_endo_ base, and LV_epi_ base. Overall, 725 restitution curves were analyzed, 74% of slopes had a maximum slope of activation recovery interval (ARI) restitution (*S*_max_) > 1 (*P* < 0.001); mean *S*_max_ = 1.76. APD was shorter in the LV_epi_ compared with LV_endo_, regardless of pacing site (30-ms difference during RV_endo_ pacing, 25-ms during LV_endo_, and 48-ms during LV_epi_; 50th quantile, *P* < 0.01). Basal LV_epi_ pacing resulted in a significant transmural gradient of RT (77 ms, 50th quantile: *P* < 0.01), due to loss of negative transmural AT-APD coupling (mean slope 0.63 ± 0.3). No significant transmural gradient in RT was demonstrated during endocardial RV or LV pacing, with preserved negative transmural AT-APD coupling (mean slope −1.36 ± 1.9 and −0.71 ± 0.4, respectively). Steep ARI restitution slopes predominate in the normal ventricle and dynamic ARI; RT gradients exist that are modulated by the site of activation. Epicardial stimulation to initiate ventricular activation promotes significant transmural gradients of repolarization that could be proarrhythmic.

## Abstract (precis)

*Spatial variation of restitution in relation to varying stimulus site is poorly defined in the intact human heart. Repolarization gradients were shown to be dependent on site of activation with epicardial stimulation promoting significant transmural gradients. Steep restitution slopes were predominant in the normal ventricle*.

## NEW & NOTEWORTHY

*Spatial variation of restitution in relation to varying stimulus site is poorly defined in the intact human heart. Repolarization gradients were shown to be dependent on site of activation with epicardial stimulation promoting significant transmural gradients. Steep restitution slopes were predominant in the normal ventricle*.

cardiac restitution describes the dynamic interaction between activation time (AT), action potential duration (APD), and repolarization time (RT) in relation to a given extrastimulus cycle length or diastolic interval (DI). It is hypothesized that a steep APD restitution slope may be proarrhythmic (22), although steep restitution curves have not been directly linked to an increased cardiac risk (15, 18, 34). However, intact whole heart ventricular restitution dynamics remain of great interest because they modulate the spatial dispersion of RT during heart rate changes. The spatiotemporal organization of AT and RT in response to an extrastimulus may, therefore, play an important role in the development of arrhythmogenesis (8). Spatial dispersion of RT is dependent on how APD varies spatially along the pathway of activation. The AT-APD interaction is often represented as a plot representing various AT and APD moments measured throughout the ventricle during the same beat. This slope between AT and APD is referred to as AT-APD coupling (24, 61). If the AT-APD slope is negative, APD decreases along the pathway of activation, limiting the dispersion of RT, while a positive AT-APD slope enhances the dispersion of RT.

The spatial variation in restitution properties and AT-APD coupling in the intact human heart has largely only been studied in situations where global AT is relatively short, such as in sinus rhythm or right ventricular (RV) apical pacing, when early engagement of the Purkinje network occurs (52, 62). This homogenizes total AT, while maintaining a normal endocardial-to-epicardial activation sequence. However, ventricular arrhythmias are frequently triggered by non-Purkinje-related premature beats (29), which may modulate repolarization gradients. Little is known about the role of varying the activation site in relation to the spatial restitution and AT-APD coupling properties of the RV and left ventricle (LV). In the intact human ventricle, negative coupling between AT-APD has been demonstrated during RV apical pacing (24, 52, 61). However, to continually maintain negative AT-ARI (activation recovery interval) coupling for varying stimulus sites, and, therefore, limit RT dispersion, regional APD would have to adapt to varying AT sequences. Regional variations in APD have been shown in animal studies using ventricular wedge preparations, where transmural and apicobasal dispersion of APD and total RT have been demonstrated (3, 5, 31). Whether these regional variations in APD are present in the intact human myocardium, and whether they adapt to varying stimulus site to maintain negative AT-APD coupling, or are fixed to only homogenize total RT in response to the normal activation sequence, remains unclear.

This study aimed to investigate the effect of varying the stimulus site on the spatial apicobasal and transmural properties of AT, APD, and RT, during restitution studies in patients with structurally normal hearts. We also sought to evaluate the effect of this on AT-APD coupling and the steepness of the APD-restitution slope. Our findings suggest that APD does not adapt when varying the stimulus site to the LV epicardium and basal LV endocardium, resulting in significant repolarization gradients, due to loss of negative AT-APD coupling (22, 36, 41, 45).

## METHODS

### 

#### Study population.

Ten patients (mean age was 45 ± 15 yr, 6 women) with structurally normal hearts who were due to undergo diagnostic electrophysiology study (EPS) for investigation of palpitations or ablation for supraventricular tachycardia were enrolled into the study. Studies were performed in the postabsorptive state under minimal conscious sedation. Six patients underwent ablation to the slow pathway as part of their clinical procedure, in the remaining patients EPS alone was conducted. All patients had normal resting electrograms with no evidence of latent preexcitation, normal echocardiograms, and normal cardiac examinations. All anti-arrhythmic drugs were stopped for 5 days before procedure. The study was approved by the local ethics committee and conformed to the declaration of Helsinki. All patients gave informed consent. The research restitution protocol began after a 20-min period of resting sinus rhythm at the end of clinical EPS.

#### Intracardiac recording.

**Fig. 1.**
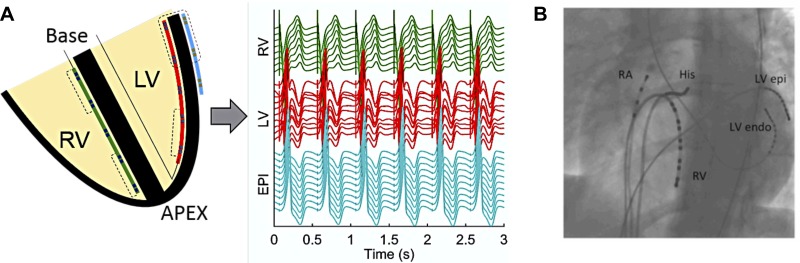
Orientation of catheters in the heart for recording. *A*: schematic showing positioning of catheters in an apicobasal orientation in the left ventricle (LV) and right ventricle (RV) endocardium (endo), and transmurally across the lateral base of the LV epicardium (EPI) via the CS, with corresponding unipolar electrograms recorded. *B*: catheter positions were checked via fluoroscopy to ensure adequate apicobasal and transmural apposition. RA, right atrium; His, bundle of His.

Following clinical EPS or after any ablation was performed, a decapolar catheter (Response; St. Jude Medical, Minnetonka, MN) was placed in the RV endocardium (RV_endo_) for recording in an apicobasal orientation (Fig. 1). A steerable decapolar catheter (Inquiry; St. Jude Medical, Minnetonka, MN) was placed on the lateral wall of the LV endocardium (LV_endo_) for recording in an apicobasal orientation in the LV via the retro-aortic approach and a steerable decapolar catheter (Inquiry; St. Jude Medical) was placed on the epicardium of the LV (LV_epi_) via the lateral cardiac vein via the coronary sinus (CS) for recording transmurally across the LV wall (Fig. 1). Transmural apposition of the catheters was checked by left anterior oblique and right anterior oblique fluoroscopy (Fig. 1*B*).

For the research study, programmed electrical stimulation was performed at a pulse width of 2 ms and stimulus strength of twice the diastolic threshold. Restitution curves were performed by pacing in three separate regions within the heart: the RV apex, the LV_endo_ at the base, and the LV_epi_ at the base, with recording made apicobasally in the RV and LV and transmurally at the base of the LV (Fig. 1).

#### Restitution protocol.

Steady state was achieved by pacing at basic cycle length of 600 ms for 3 min. Following this, an S_1_-S_2_ restitution protocol was performed, beginning with an extra stimulus (S_2_) at 1,000 ms. The S_1_-S_2_ coupling interval was then decremented in 50-ms steps until an S_2_ of 400 ms, then by 20-ms intervals between 300 and 400 ms, and thereafter in 5-ms steps, until effective refractory period (ERP) of the tissue. At ERP an S_2_ stimulus at 10 ms + ERP was applied followed by further decrementing S_2_ in steps of 2 ms to confirm ERP.

#### AT and RT evaluation.

**Fig. 2.**
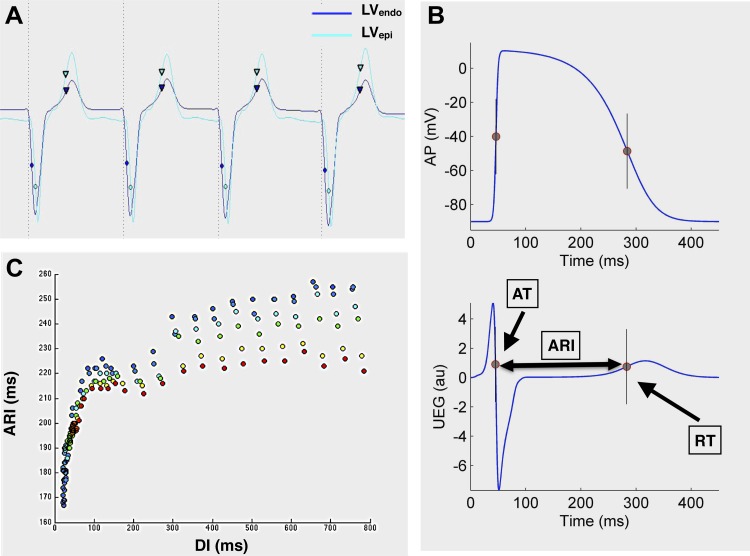
Analysis of cardiac restitution studies. *A*: example of simultaneous transmural unipolar electrogram recording at the LV_endo_ base and LV_epi_ base during LV endocardial restitution. Activation moments (circles) and repolarization moments (inverted triangles) are shown. *B*: the Wyatt method was used to determine activation time (AT) and repolarization time (RT), with activation recovery interval (ARI) taken as a surrogate marker of APD. UEG, unipolar electrogram; au, arbitrary units. *C*: example of an ARI restitution curve of one patient, from a series of catheter poles in the RV. Each pole is represented by a different color.

Unipolar electrograms were filtered at 0.05–500 Hz and recorded at a sampling rate of 2,000 Hz (Bard Clearsign, CR Bard, NJ) (Fig. 2*A*). Local AT was calculated as the interval from pacing stimulus to the minimum of the first derivative of the unipolar QRS complex (dV/d*t*_min_) (Fig. 2, *A* and *B*) (10). Local RT was defined using the Wyatt method (10), as the maximum the first derivative of the unipolar T-wave (dV/d*t*_max_, Fig. 2, *A* and *B*). The Wyatt method was chosen because of its firm theoretical and experimental basis. It is established as the most accurate surrogate measure of local RT in the unipolar electrogram in experimental (10, 25, 59) and theoretical studies (43, 49) and has been shown to be independent of activation sequence (25). ARI was used as a surrogate marker of APD and was calculated as ARI = RT − AT (Fig. 2*B*) (10). DI was obtained by calculating the local cycle length as the interval from the AT after the last beat at basic cycle length S1 and AT after the premature beat S_2_ (AT_S2_ − AT_S1_) and then subtracting the median ARI for the preceding S_1_ train after discarding the first three beats. This was done to minimize the effect of ARI dynamics on DI and to obtain a reliable DI even for very short S1–S2 coupling intervals.

Drive trains that contained ectopic beats were excluded. Signals at the pacing site or where it was not possible to calculate local RT due to abnormal T-wave morphology or ST segment elevation with no clear upstroke were excluded. In four patients, CS pacing was excluded in analysis due to inadequate epicardial capture for complete restitution curves to be generated. Recordings characterized by a signal-to-noise ration lower than 13 dB were discarded.

#### Data analysis.

Unipolar electrograms were processed offline using MATLAB_R2014a (The MathWorks, Natick, MA, 2014). AT, ARI, and RT were calculated semiautomatically using a custom-made MATLAB interface, as in previous studies (39, 58), and all markers were independently checked manually by two separate reviewers (NS and MO). Maximum slope of ARI restitution (*S*_max_) was characterized by plotting ARI vs. DI for each restitution curve and defined as the steepest slope using piecewise linear regression on a moving 40-ms window, as previously described (55).

For the analysis of regional differences within the heart, basal measurements were taken as the mean of the last four catheter poles located at the base of the RV_endo_ and LV_endo_ within each patient (Fig. 1). Apical measurements were taken as the mean of the last four catheter poles located at the apex of the heart in the RV_endo_ and LV_endo_ in each patient. Transmural comparisons could only be made across the base of the lateral LV, comparing the mean of the four basal LV endocardial catheter poles and four catheter poles placed in the epicardium of the basal LV via the CS. This was because it was not possible to consistently record from the apex of the epicardium of the heart via the coronary veins due to variation in anatomy. Regional AT, ARI, and RT restitution curves, taking into account the data from all patients, were then assessed using Lowes regression with 95% confidence intervals, to take into account the triphasic shape of the typical restitution curve, as described by Franz (18) (Fig. 2*C*). Differences between different restitution curves were analyzed using quantile regression across the whole of the restitution curve, with statistical significance inferred only if *P* < 0.05 across the entire restitution curve. To check that the results were independent from the chosen approach, data were also analyzed on a patient-by-patient basis. Results from Lowes regression and patient-by-patient analysis were highly consistent for each cardiac interval and pacing site.

The AT-ARI relationship was evaluated using the linear regression slope of the plot of AT vs. ARI at a cycle length of 600 ms. Slopes were calculated for all three different pacing sites within each individual patient. Global AT-ARI coupling was assessed from the linear regression slope of all electrograms recorded within the heart. Regional coupling was assessed from the regression slope in the RV_endo_ and LV_endo_ of all electrograms recorded within the RV and LV, respectively, and transmurally from the electrograms recorded across the basal lateral wall of the LV (Fig. 1*B*).

#### Statistical analysis.

Statistical analysis was performed using R statistical software (R version 3.0.3, R Foundation for Statistical Computing, Vienna) (44a). Continuous parametric data are presented as means ± SD, or, in the case of nonparametric data, median, unless otherwise specified. One-sampled Student's *t*-test was used to assess the statistical significance of *S*_max_ being >1 and AT/ARI slope being negative. Comparison between restitution slopes in the RV_endo_, LV_endo_, and LV_epi_ was performed using one-way ANOVA with Tukey post hoc correction.

## RESULTS

### 

#### Shape of the ARI restitution curve and maximum slope.

The shape of the restitution curve across a series of six RV catheter poles during RV pacing for a representative study patient is shown in Fig. 2*C*. The triphasic nature of the curve was a consistent finding in all patients, and, therefore, Lowes regression was used to analyze restitution curve data across all patients and interregional AT, ARI, and RT differences in relation to stimulus site.

**Fig. 3.**
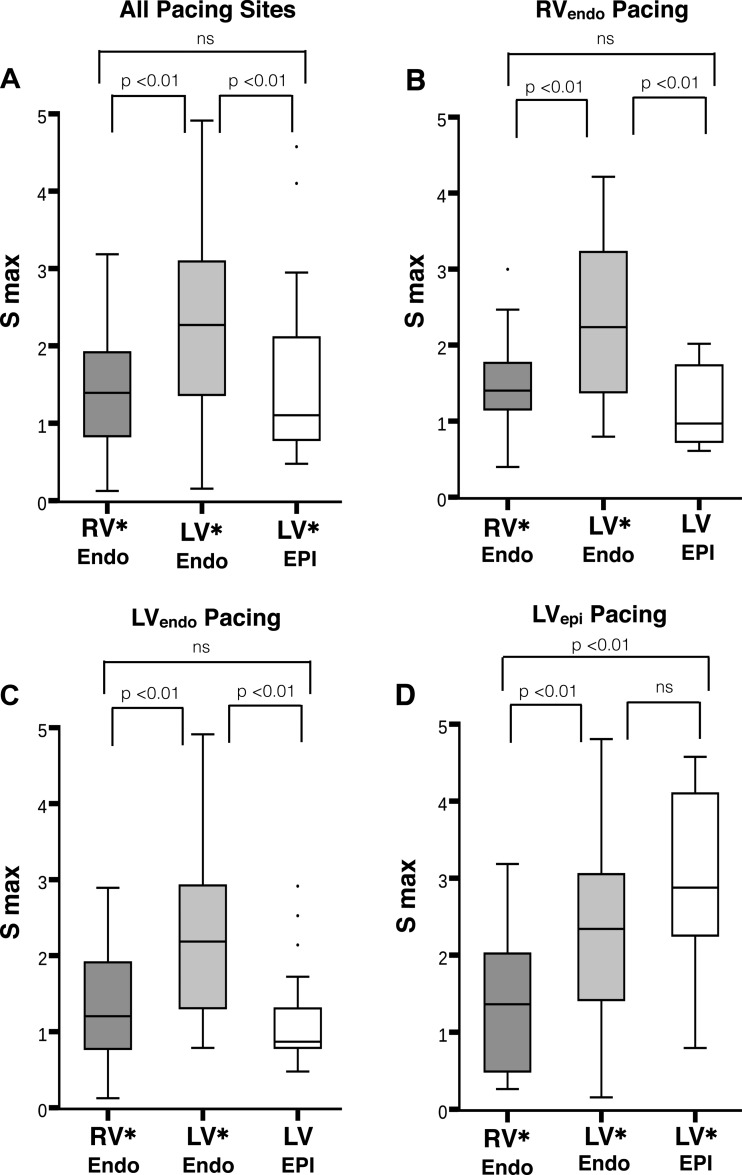
Boxplots of *S*_max_ for all patients, with *S*_max_ on *y*-axis and recording region on the *x*-axis. *A*: all recorded *S*_max_ in the endocardium (Endo) of the right ventricle (RV), left ventricle (LV), and epicardium of the left ventricle (Epi), regardless of pacing site. *B*, *C*, and *D*: *S*_max_ for RV_endo_, LV_endo_, and LV_endo_ pacing, respectively. The median *S*_max_ was >1 in all instances. Bars above the graphs represent ANOVA comparisons of statistical difference between regional *S*_max_, based on pacing site. *P < 0.05 for regional *S*_max_ >1, based on one-sampled *T*-test.

A total of 725 individual restitution slopes complied with data quality criteria and were analyzed: 290 slopes from the RV, 290 slopes from the LV, and 145 slopes from the epicardium; 74% of slopes had a maximum slope > 1 (*P* < 0.001), with a mean *S*_max_ = 1.76 and median *S*_max_ = 1.54. Figure 3 displays the distribution of *S*_max_ grouped by recording and pacing sites. The median *S*_max_ was >1 in all instances. *S*_max_ was significantly >1 (*P* < 0.05) for all regions, except in the LV_epi_, when performing restitution from the RV apex (*P* = 0.26) and LV basal endocardium (*P* = 0.13). *S*_max_ was greater (Fig. 3*A*) in the LV_endo_ (mean *S*_max_ = 2.2) compared with the RV_endo_ (mean *S*_max_ = 1.4, *P* < 0.01) and LV_epi_ (mean *S*_max_ = 1.5, *P* < 0.01), and there was no significant difference in the mean slope between the RV_endo_ and LV_epi_, irrespective of pacing site. The same pattern was noted with RV apical and LV basal pacing with steeper *S*_max_ throughout the LV_endo_ compared with the RV_endo_ and LV_epi_ (Fig. 3, *B* and *C*). During epicardial LV pacing, *S*_max_ was steepest in the LV_epi_ and LV_endo_, with no significant difference between the regions on ANOVA (*P* = 0.26, mean *S*_max_ 2.9 vs. 2.2), but significantly greater *S*_max_ for both regions compared with the RV (mean *S*_max_ 1.3, *P* < 0.01 for both regions). Thus there was no consistent relationship between steepness of the restitution curve and distance from pacing site.

#### Activation pattern and AT restitution.

**Fig. 4.**
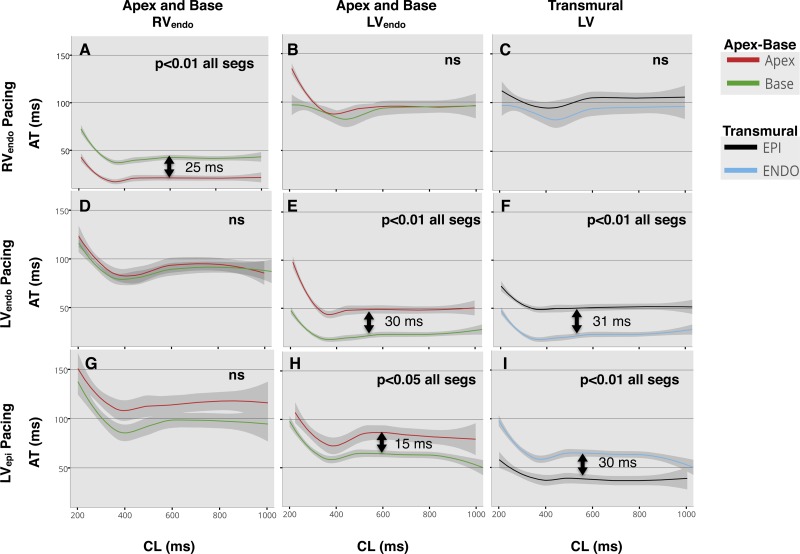
Lowes regression of activation time (AT) restitution in all patients, with 95% confidence interval. Pacing site for each row is shown: endocardium of the right ventricle (RV_endo_; *A–C*), endocardium of the left ventricle (LV_endo_; *D–F*), and LV_epi_ (*G–I*). Comparisons are made between apex and base in the RV_endo_ (*A*, *D*, and *G*), LV_endo_ (*B*, *E*, and *H*), and transmurally across the LV base (*C*, *F*, and *I*), represented by each column heading (*top*). Significant differences were assumed if quantile regression was <0.05 across the whole of the restitution curve, and statistical significance is shown inset within the graphs. Arrows with time in miliseconds (ms) represent difference at the 50th quantile, where curves were significantly different. ns, No significance; CL, cycle length.

Differences in apicobasal and transmural AT are shown in Fig. 4. The regional pattern of AT for each one of the stimulus sites can be followed by inspection of each line of panels in Fig. 4.

Lowes regression of AT restitution when pacing from the RV apex (Fig. 4, *A–C*) showed initial activation of the apex and then base of the RV with minimal delay, followed by almost simultaneous activation of the apex and base of the lateral wall of the LV, and finally epicardial activation. AT restitution when pacing the base of the LV_endo_ (Fig. 4, *D–F*) had the expected earliest activation of the LV base, followed by delayed conduction to the LV apex and transmurally to the basal LV_epi_, followed by almost simultaneous activation of the RV at the base and apex. Pacing from the basal LV_epi_ (Fig. 4, *G–I*) resulted in earliest activation of the epicardium of the LV, followed by basal endocardial LV activation. This was followed by activation of the LV apex and then activation of the RV initially at the base and then the apex. All slopes displayed the expected finding of AT restitution with lengthening of AT at shorter cycle lengths close to the refractory period of the ventricle (Fig. 4).

#### Regional ARI restitution and dispersion of ARI.

**Fig. 5.**
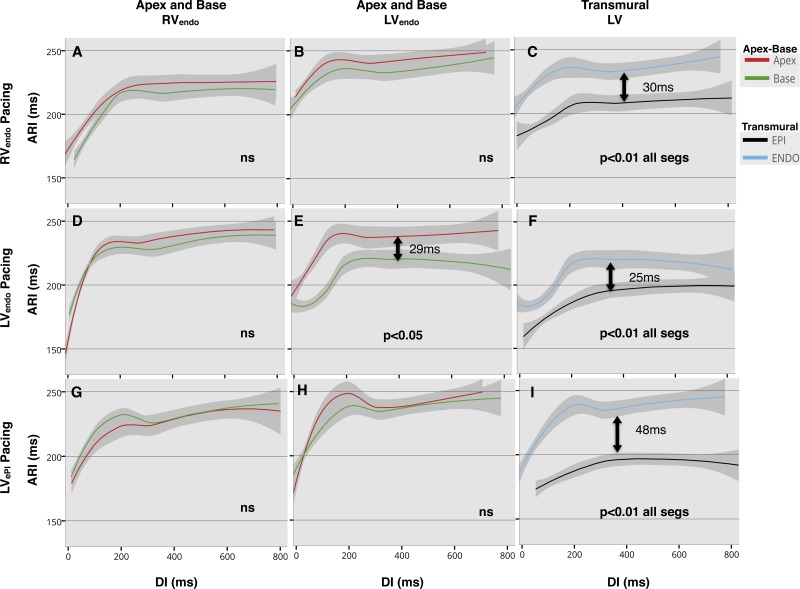
Lowes regression of activation recovery interval (ARI) restitution in all patients, with 95% confidence interval. Pacing site for each row is shown: endocardium of the right ventricle (RV_endo_; *A–C*), endocardium of the left ventricle (LV_endo_; *D–F*), and LV_epi_ (*G–I*). Comparisons are made between apex and base in the RV_endo_ (*A*, *D*, and *G*), LV_endo_ (*B*, *E*, and *H*), and transmurally across the left ventricular (LV) base (*C*, *F*, and *I*), represented by each column heading (*top*). Significant differences were assumed if quantile regression was <0.05 across the whole of the restitution curve, and statistical significance shown inset within the graphs. Arrows with time in miliseconds (ms) represent difference at the 50th quantile, where curves were significantly different. There is a consistent transmural difference in ARI, regardless of pacing site (*C*, *F*, and *I*), with a significant LV apicobasal difference in ARI on LV_endo_ pacing only (*E*). DI, diastolic interval; ns, no significance.

In the RV, restitution curves showed a trend toward shorter ARI at the base than at the apex (Fig. 5, *A*, *D*, and *G*), but no significant apicobasal differences were observed, regardless of the site at which pacing was conducted. In the LV_endo_, restitution curves (Fig. 5, *B*, *E*, and *H*) again showed a trend toward shorter ARI at the base compared with the apex, regardless of pacing site, with significant apicobasal differences only when pacing the LV base endocardially (ARI at the base was 29 ms shorter than at the apex, *P* < 0.05 at 50th quantile, Fig. 5*E*). Transmural differences in ARI restitution from all three different pacing sites are shown in Fig. 5, *C*, *F*, and *I*. There was a significant transmural difference in ARI across the whole restitution curve, regardless of pacing site (*P* < 0.01 for all curves), with shorter ARI epicardially than endocardially. The greatest transmural difference in ARI was measured when pacing the LV base epicardially (48 ms at 50th quantile, Fig. 5*I*), while it was lower when pacing endocardially, being 25 and 30 ms when pacing the LV and the RV, respectively (Fig. 5, *F* and *C*, respectively).

#### Regional RT restitution and dispersion of RT.

**Fig. 6.**
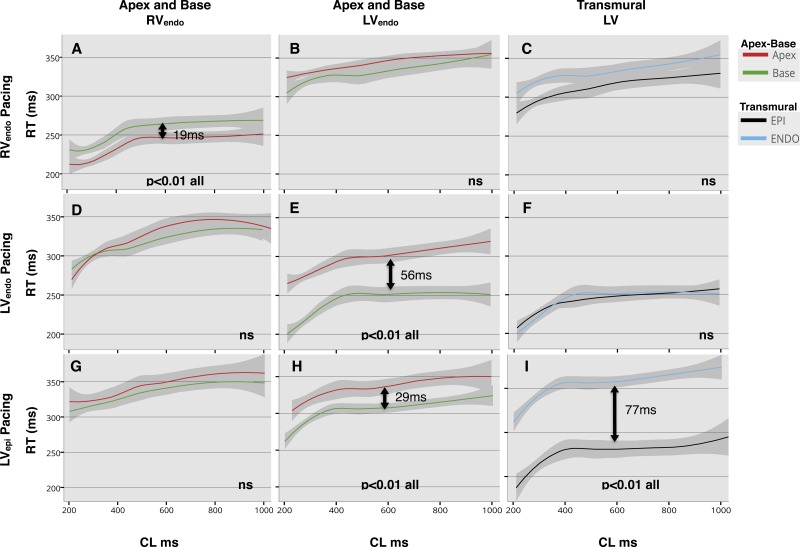
Lowes regression of repolarization time (RT) restitution in all patients, with 95% confidence interval. Pacing site for each row is shown: endocardium of the right ventricle (RV_endo_; *A–C*), endocardium of the left ventricle (LV_endo_; *D–F*), and LV_epi_ (*G–I*). Comparisons are made between apex and base in the RV_endo_ (*A*, *D*, and *G*), LV_endo_ (*B*, *E*, and *H*), and transmurally across the left ventricular (LV) base (*C*, *F*, and *I*), represented by each column heading (*top*). Significant differences were assumed if quantile regression was <0.05 across the whole of the restitution curve, and statistical significance is shown inset within the graphs. Arrows with time in milliseconds (ms) represent difference at the 50th quantile, where curves were significantly different. There is a significant apicobasal gradient in RT on RV and LV pacing (*A* and *E*), while CS pacing creates a significant transmural gradient in RT (*I*), as well as an LV apicobasal gradient in RT (*H*). CL, cycle length; ns, no significance.

In the RV_endo_, RT showed a small difference across the entire restitution curve when pacing from the RV apex only (*P* < 0.01); with a 19-ms difference in RT at the apex compared with the base at the 50th quantile (Fig. 6*A*).

In the LV_endo_, significant apicobasal RT differences were only seen when pacing at the LV base both endocardially and epicardially (Fig. 6, *E* and *H*), with RT shorter at the base and longer at the apex. At the 50th quantile, apicobasal RT differences were 56 and 29 ms, when pacing the LV endocardially and epicardially, respectively.

Significant LV transmural differences in RT were seen when pacing at the basal epicardium of the LV (Fig. 6*I*, *P* < 0.01). At the 50th quantile, the epicardial RT was 77 ms shorter than the endocardium at the base of the LV. There was no significant transmural difference in RT during endocardial pacing from the RV apex and LV base (Fig. 6, *C* and *F*).

#### AT-ARI coupling.

Table 1 shows the mean and SE of AT-ARI coupling measured in all patients during pacing at basic cycle length of 600 ms. When evaluating global AT-ARI coupling over all measured cardiac sites within the heart, no negative correlation was observed for pacing at RV apex and the LV basal endocardium and epicardium, indicating a loss of negative global AT-ARI coupling during pacing at these sites.

**Table 1.** Global and regional AT/ARI coupling slope gradient by varying pacing site

	Global Coupling Slopes	RV_endo_ Coupling Slopes	LV_endo_ Coupling Slopes	LV Transmural Coupling Slopes
RV_endo_ pacing	−0.03 ± 0.1	−0.61 ± 0.3	−0.42 ± 0.9	−1.36 ± 1.9
LV_endo_ pacing	0.14 ± 0.2	0.20 ± 0.4	0.81 ± 0.3	−0.71 ± 0.4
LV_epi_ pacing	0.4 ± 0.2	0.04 ± 0.2	0.63 ± 0.4	0.63 ± 0.3

Values are means ± SE.

RV, right ventricle; LV, left ventricle; epi, epicardial; endo, endocardial.

During RV pacing, regional negative AT-ARI coupling was preserved in the RV (−0.61 ± 0.3), in the LV (−0.42 ± 0.9), and transmurally (−1.36 ± 1.9). During pacing at the basal LV_endo_, negative AT-ARI coupling was only present transmurally (−0.71 ± 0.4). During pacing at the LV_epi_, there was a loss of negative regional AT-ARI coupling, indicating that ARI increased with increasing AT.

## DISCUSSION

This is the first study to systematically examine the effects of stimulation site on the RV endocardial, LV endocardial, and LV epicardial conduction-repolarization dynamics in the intact human heart. The major finding of this study is that intrinsic heterogeneity of APD within the ventricle promotes a dispersion of repolarization, depending on the stimulus site, due to failure of APD to adapt to varying activation sequences. This may play an important role in arrhythmogenicity. Second, we demonstrate the presence of negative AT-ARI coupling is dependent on activation sequence. Third, we demonstrate in patients with structurally normal hearts and low risk of ventricular arrhythmia that an *S*_max_ > 1 predominates, regardless of stimulation site. Finally, although often studied and modeled in a monotonic exponential, our data confirm that the human restitution curve is triphasic in nature, as previously described (20, 21), consisting of a steep early phase, followed by a “hump” or “dip”, and lastly a plateau.

### 

#### Intrinsic ARI heterogeneity synchronizes ventricular repolarization during normal activation, but fails to adapt to ectopic stimulation, resulting in loss of negative AT-ARI coupling.

The presence of intrinsic APD heterogeneity within the ventricle has been shown in studies demonstrating electrical heterogeneity of channel expression (2, 3, 5, 11, 14, 19, 26, 31, 42a, 48, 51). A greater density of the slow-component delayed rectifier K^+^ current channels is thought to be present at the base of the LV compared with the apex, resulting in shorter APD at the base (1). In addition, greater transient outward K+ current channel density in the epicardium of the heart may cause a shorter APD in the epicardium than the endocardium (6, 30). These apicobasal and transmural gradients of APD have also been demonstrated in human studies (13, 17, 53), and our findings are consistent with both in vitro and in vivo observations in showing a small apicobasal gradient in ARI in the RV and LV and a larger (30-ms) LV transmural ARI gradient on RV apical pacing (Fig. 5, *A–C*).

The function of this heterogeneity in ARI may be to compensate for the normal apicobasal and endocardial-to-epicardial activation sequence within the ventricle to homogenize total RT, as demonstrated by the lack of significant dispersion of repolarization during RV pacing (Fig. 6, *A–C*) and, therefore, the presence of negative regional AT-ARI coupling (Table 1). This is consistent with previously published data in human and animal studies (6, 13, 19, 26, 32, 40, 42, 47, 48, 53). Thus the heterogeneity in ARI results in regional repolarization wavefronts that are opposite to the wavefront of activation, consistent with the concept of negative AT/ARI coupling, as shown in Table 1, and consistent with previous in vivo experimental data (23, 52). Our study shows that this homogenization of total repolarization within localized regions of the heart occurs over a range of cycle lengths and may be protective against localized reentry during normal physiological working parameters of the ventricle, particularly sinus tachycardia.

By varying the stimulus site, however, our data show that local ARI does not adapt to a change in activation sequence (Fig. 5, *D–I*), with regional differences in ARI being maintained and inverse coupling, therefore, disrupted (Table 1). When basal LV_endo_ and LV_epi_ are paced, there is loss of global and regional negative AT-ARI coupling (Table 1) as a result of ARI increasing with AT. This may be partly due to the relatively fixed nature of regional APD, which failed to adapt to varying the stimulus site, to alterations in the activation of the Purkinje network, or to changes in local electrotonic forces at different stimulus sites. The resultant effect of this loss of coupling is a further shortening of local ARI at the early activating stimulus site compared with RV pacing (Fig. 5, *B* and *C*); with 29-ms apicobasal dispersion in ARI (Fig. 5*E*) during LV_endo_ pacing and a 48-ms transmural ARI dispersion during LV_epi_ pacing (Fig. 5*I*), which amplifies regional ARI differences and enhances RT dispersion (Fig. 6, *E* and *I*).

The failure of AT-ARI coupling to adapt to a varying stimulus site by continuing to be negative brings into question whether negative coupling is a true electrotonic phenomenon, or a function of fixed differential channel expression within the ventricle, due to the effect of cardiac memory, for the normal activation sequence (26, 42, 47, 48). It is well known, for instance, that short- and long-term pacing can modulate regional APD within the heart, particularly in the epicardium. This change in APD secondary to a new activation pattern (46, 47) potentially prevents dangerous localized repolarization gradients from occurring. However, ectopic beats that suddenly alter the normal activation sequence, as in this study, may have a greater effect on RT dispersion because early initial APD adaptation does not occur. Thus the location of ectopic or pacing beats within the heart may play as important a factor in arrhythmogenicity as its timing, by creating regional windows of dispersed repolarization, as demonstrated in our study (Fig. 6, *E*, *H*, and *I*). This has important implications both with regard to determining whether ventricular ectopy is benign and in relation to where ventricular tachycardia stimulation protocols are currently performed.

#### Steepness and shape of the restitution slope in relation to arrhythmogenesis.

Although restitution slopes with *S*_max_ > 1 have been theoretically implicated in an increased risk of ventricular arrhythmia (27, 36, 41, 44), *S*_max_ > 1 has also been demonstrated in human ventricles (20, 21, 35, 55). Additionally, in patients at high risk of ventricular arrhythmia *S*_max_ > 1 has not been shown to be associated with long-term prognostic outcome or risk of T-wave alternans (15, 34, 63). Our study is the first study using contact electrograms to document that ARI restitution curves with *S*_max_ > 1 predominate in the normal intact human myocardium, regardless of pacing site, and shows the importance of performing a restitution curve until ERP to measure restitution at short DIs that invoke AT restitution. Yue et al. (62) and Taggart et al. (55) have previously found steep restitution slopes in human hearts. However, these studies were not entirely from a cohort of patients with structurally normal hearts. Additionally, Yue et al. (62) showed that the maximum restitution slope was >1 in 25% of all sites compared with 74% of sites in our study. Similar to our study, they also showed that *S*_max_ in the LV was greater than *S*_max_ in the RV (62), perhaps due to muscle and sodium channel density differences. This indicates that other factors also play an important role in arrhythmic risk. Due to the rapid adaptive shortening of APD (21) and the reciprocal lengthening in DI (18), steep restitution slopes may actually be protective in the normal ventricles during sinus tachycardia and closely coupled ventricular ectopy, as subsequent beats at a similar cycle length would “run-off” to a flatter portion of the restitution curve, as has been previously suggested (19, 20).

Our restitution slopes were also triphasic in morphology, as has previously been demonstrated (20, 21), and modeling studies should take this into account (37). The “hump” or second phase of the ventricular restitution curve may be governed by the kinetics of the L-type calcium channel and the delayed rectifier potassium channels (18), the expression of which is known to be heterogeneous throughout the heart (1). The behavior of this phase of the restitution curve, close to the steep portion of the curve and the refractory period, may also play an important role in dispersion of repolarization and arrhythmogenesis.

#### Scientific and clinical implications.

The finding that intrinsic heterogeneity of APD within the intact human ventricle promotes a significant dispersion of repolarization, depending on the activation pattern, has important implications. It is intriguing to find that epicardial stimulus should create a significant transmural dispersion in repolarization in this intact human heart study and in the study of Boukens et al. (6), while an endocardial activation pattern does not. Dispersion of repolarization is an important factor in the genesis of ventricular arrhythmia (7), especially in cases of functional reentry. Although regional dispersion of repolarization alone is not sufficient to establish a reentrant circuit (12, 38), it is a recognized substrate for the development of reentry (8). This raises questions as to whether epicardial ectopy may be more arrhythmogenic than ectopy from endocardial sites. Additionally, in a subset of patients, epicardial pacing during cardiac resynchronization therapy has been shown to be arrhythmogenic (9, 17, 33, 50, 56), particularly in the acute phase following implant, before adaptation occurs. This may potentially be explained by the fact that LV epicardial pacing promotes a significant transmural repolarization gradient when the ventricle was previously exposed to a normal endocardial-to-epicardial activation sequence, as has been demonstrated in human heart wedge preparation studies (6) and in this study. These hypotheses require further validation and investigation in future studies.

#### Limitations.

Data were confined to multielectrode unipolar contact catheter recordings in the RV_endo_, LV_endo_, and LV_epi_ in patients admitted for EPS, as opposed to global mapping, therefore limiting the characterization of AT, ARI, and RT spatial patterns. This was because it is not possible to use technologies that offer a better spatial view and resolution, such as noncontact mapping, multielectrode socks (54), or optical mapping, during minimally invasive in vivo human studies. Furthermore, significant assumptions, limitations, and inaccuracies may occur with the use of noncontact mapping systems (16). Although great care was taken to ensure fluoroscopic transmural apposition of the catheters, it should be noted that true fiber-orientated transmural measurements, akin to wedge preparations (60) or plunge electrode recordings (53), were not possible. However, we did consistently record shorter ARIs epicardially in our patients, and the finding of no significant difference in RT transmurally during endocardial activation sequences would support the accuracy of our catheter positioning, as it is consistent with in vitro studies (6).

It was not possible to directly test whether repolarization dispersion enhanced by ventricular stimulation could serve as an arrhythmogenic substrate, because ventricular tachycardia/fibrillation induction was not included in the study protocol for ethical reasons. Finally, the slope of the S1–S2 APD-restitution protocol has been reported to be higher than that obtained with a dynamic protocol (28), which may be more accurate in revealing propensity toward the development repolarization alternans and cardiac instability.

#### Conclusion.

Steep ARI restitution slopes predominate in the normal ventricle and dynamic ARI; RT gradients exist that are modulated by the site of activation.

Basal epicardial and endocardial LV stimulation to initiate ventricular activation promotes significant transmural and apicobasal gradients of repolarization due to failure of APD to adapt to varying stimulus sites, with resultant loss of regional AT-APD coupling.

## GRANTS

This work was supported University College London Hospitals Biomedicine National Institute for Health Research. N. T. Srinivasan was supported by a British Heart Foundation Clinical Research Training Fellowship (FS/14/9/30407). M. Orini was supported by Marie Curie Fellowship (IEF-2013). P. D. Lambiase and P. Taggart were supported by the Medical Research Council (G0901819).

## DISCLOSURES

No conflicts of interest, financial or otherwise, are declared by the author(s).

## AUTHOR CONTRIBUTIONS

N.T.S., M.O., R.B.S., R.P., R.B., E.R., A.W.C., P.T., and P.L. conception and design of research; N.T.S., M.O., R.B.S., R.P., F.K., O.R.S., G.B., R.B., E.R., S.A., A.W.C., M.L., and P.L. performed experiments; N.T.S. and M.O. analyzed data; N.T.S., M.O., R.B.S., R.P., F.K., O.R.S., G.B., R.B., E.R., S.A., A.W.C., M.L., P.T., and P.L. interpreted results of experiments; N.T.S. and M.O. prepared figures; N.T.S., M.O., R.B.S., R.P., F.K., O.R.S., G.B., R.B., E.R., S.A., A.W.C., M.L., P.T., and P.L. drafted manuscript; N.T.S., M.O., R.B.S., R.P., F.K., O.R.S., G.B., R.B., E.R., S.A., A.W.C., M.L., P.T., and P.L. edited and revised manuscript; N.T.S., M.O., R.B.S., R.P., F.K., O.R.S., G.B., R.B., E.R., S.A., A.W.C., M.L., P.T., and P.L. approved final version of manuscript.
